# Show Me Your Rump Hair and I Will Tell You What You Ate – The Dietary History of Muskoxen (*Ovibos moschatus*) Revealed by Sequential Stable Isotope Analysis of Guard Hairs

**DOI:** 10.1371/journal.pone.0152874

**Published:** 2016-04-20

**Authors:** Jesper Bruun Mosbacher, Anders Michelsen, Mikkel Stelvig, Ditte Katrine Hendrichsen, Niels Martin Schmidt

**Affiliations:** 1 Arctic Research Centre, Department of Bioscience, Aarhus University, Frederiksborgvej 399, DK-4000 Roskilde, Denmark; 2 Department of Biology, University of Copenhagen, Universitetsparken 15, DK-2100 Copenhagen Ø, Denmark; 3 Center for Permafrost, University of Copenhagen, Øster Voldgade 10, DK-1350 Copenhagen K, Denmark; 4 Copenhagen Zoo, Roskildevej 38, DK-2000 Frederiksberg, Denmark; 5 Norwegian Institute of Nature Research, Høgskoleringen 9, NO-7034 Trondheim, Norway; University of Florida, UNITED STATES

## Abstract

The nutritional state of animals is tightly linked to the ambient environment, and for northern ungulates the state strongly influences vital population demographics, such as pregnancy rates. Continuously growing tissues, such as hair, can be viewed as dietary records of animals over longer temporal scales. Using sequential data on nitrogen stable isotopes (δ^15^N) in muskox guard hairs from ten individuals in high arctic Northeast Greenland, we were able to reconstruct the dietary history of muskoxen over approximately 2.5 years with a high temporal resolution of app. 9 days. The dietary chronology included almost three full summer and winter periods. The diet showed strong intra- and inter-annual seasonality, and was significantly linked to changes in local environmental conditions (temperature and snow depth). The summer diets were highly similar across years, reflecting a graminoid-dominated diet. In contrast, winter diets were markedly different between years, a pattern apparently linked to snow conditions. Snow-rich winters had markedly higher δ^15^N values than snow-poor winters, indicating that muskoxen had limited access to forage, and relied more heavily on their body stores. Due to the close link between body stores and calf production in northern ungulates, the dietary winter signals could eventually serve as an indicator of calf production the following spring. Our study opens the field for further studies and longer chronologies to test such links. The method of sequential stable isotope analysis of guard hairs thus constitutes a promising candidate for population-level monitoring of animals in remote, arctic areas.

## Introduction

Quantity and quality of forage is decisive for herbivore body condition and health, and may in turn affect vital population demographics, such as pregnancy rates and survival [1]. The ability to track animal diets as an indicator of changes in the environmental conditions, and thereby their likely consequences for animal population dynamics, are hence of great interest. Here, stable isotope analyses constitute a valuable tool for unravelling animal diets, as stable isotopes in animal tissues and excreta reflect dietary preferences, and yield insight into the environmental conditions experienced by the animal [2, 3]. Hair, in particular, offers a very promising opportunity to investigate an animal’s dietary history over extended periods [4–7]. Hairs may grow continually over extended periods of time, and remain metabolically inactive after their formation [4, 8]. Thus, hair can be regarded as an isotopic archive of the dietary preference during the time in which the tissue was formed [4], and hair segments may therefore serve as a temporally explicit record of animal diet, revealing dietary changes induced by seasonal and annual fluctuations in the ambient environment [4, 5, 7, 9]. Being able to understand the link between environmental conditions and animal diets, and thus ultimately to population dynamics, would greatly extend our understanding of the important drivers of changes in animal populations, and ultimately inform proper conservation and management initiatives. The analysis of stable isotopes in animal tissue or excreta may thus be a valuable, and often non-invasive, tool for species monitoring [10–12].

In remote regions, like the Arctic, such tools may greatly enhance the collection of valuable population monitoring data. The Arctic is currently experiencing marked changes in climate and a number of other environmental parameters [13–15], which stresses the need to reduce the existing knowledge gap on herbivore ecology in a changing Arctic [16]. In the tundra ecosystem, the muskox, *Ovibos moschatus*, plays a key role as one of few large herbivores [17, 18]. Residing in the Arctic year-round, the muskox experiences highly variable levels of forage quality and quantity, both seasonally and annually [19, 20]. As most northern ungulates, the muskox relies heavily on fat reserves built up over summer and autumn as an energy reserve during the snow-covered period [21–24]. In addition, adult females must support pregnancy and lactation through body stores until plant growth resumes in spring [23]. Hence, the productivity and survival of muskoxen is strongly influenced by the nutritional state of females in particular [22, 25]. By the end of the rutting season, adult females must have replenished their fat reserves to about 20% fat in the ingesta–free body mass in order to participate in the mating [22]. Consequently, muskoxen forage mainly on energy rich graminoids in productive wetter ecosystem types during summer, while shifting to willows, *Salix sp*., on the more barren, wind exposed mountain ridges in winter [19, 26, 27]. Access to sufficient amounts of graminoids may thus be decisive for the amount of fat and maternal body protein stores being build up during summer, which are crucial for the production of calves [28, 29]. Failure to adequately restore body fat over the summer and autumn may hence in part explain the high variability of calf production in muskoxen. Pregnancy rates can exceed 90% in wild muskox populations [30], but under more unfavorable conditions pregnancy rates are more moderate, and occasionally no calves are born at all [31–33]. As graminoids have higher δ^15^N ratios compared to willows [26, 34], we expect to find a strong seasonal pattern in the δ^15^N hair chronology, reflecting the seasonal changes in diet, with high δ^15^N during snow-free periods, and lower δ^15^N values during snow-covered winter periods. We expect that muskox productivity is closely linked to the quality and quantity of the availability of forage. Thus, changes in the environmental conditions and hence the diet may therefore influence the calf recruitment—ultimately affecting the dynamics of muskox populations [35].

Knowledge about the temporal variation in muskox diet is therefore vital to our understanding of muskox dynamics. In this study, we aim to assess the applicability of sequential stable isotope analyses of muskox guard hairs to reconstruct the intra- and inter-annual variation in muskox diet, and ultimately to assess the use of sequentially stable isotope analyses as a tool for monitoring muskoxen and other ungulates in remote areas, such as the Arctic. We successfully recreated an approximately 2.5 year dietary history of muskoxen with a high temporal resolution. Our results highlighted the method as a possible monitoring tool for animals in remote areas.

## Materials and Methods

### Study area and sample collection

Muskox hair samples were collected in Zackenberg valley in Northeast Greenland (74°28’N, 20°34’W) in October 2013. Zackenberg is located in a high arctic climate with an annual mean air temperature of -9°C and average precipitation of 261 mm, mainly falling in autumn as snow [36]. Zackenberg valley and the surrounding region of Wollaston Forland are covered by a mosaic of different vegetation types [37]. During summer, muskoxen forage mainly in the graminoid-dominated areas, but also in *Salix* snowbeds and heaths [26], while they switch to a *Salix*-dominated diet during winter [19, 27, 38]. The density of muskoxen at Zackenberg is among the highest reported for the Arctic [35]. In 1990, the total population in the Wollaston Forland region, in which Zackenberg is located, was estimated to be between 2,900 to 4,600 individuals [39]. Data from Zackenberg have revealed an increase in muskox abundance from 1996 to 2007, after which the population declined markedly [35].

The body of muskoxen is covered with long guard hairs. Guard hairs may reach a length of up to 60 cm, but the average length is about 10–15 cm [40, 41]. Unlike the wool that is shed annually, the guard hairs continue to grow over several years, and are fully grown after about 3–4 years [42]. Guard hairs from the rump, which makes up the characteristic “skirt” of muskoxen, are the longest and grow continuously year-round [42].

In connection with a GPS collaring study by the same authors, we obtained guard hair samples from a total of 10 adult tranquilized muskox cows, ages 4 years or more, randomly chosen in the Zackenberg valley. Hair samples were cut from the rump region using an electric hair clipper. The hairs were cut at the base of the skin and placed in individual zip lock polyethylene bags until processing in the laboratory.

### Guard hair sectioning and analyses of stable isotopes

In order to get a high temporal resolution of the dietary history, guard hairs were cut into 2 mm sections before being analyzed. Chronologies and growth rates of hairs from the same individual are strongly correlated [4, 7], and 40–50 guard hairs from each individual were aligned next to each other before cutting in order to obtain a sufficient amount of material in each section, and fixated in a 7% agarose gel (Electran® agarose DNA grade, VWR). Hairs were then sequentially (serially) cut into the 2 mm pieces for stable isotope analysis. The agarose gel of each section was afterwards melted away in boiling, distillated water, before the hair samples was cleaned in 96% ethanol, oven-dried at 50°C for 24 hours, and packed into tin capsules each holding between 0.3–1.0 mg of guard hair. As δ^15^N can be used to decipher diets of muskoxen [26], we analyzed the δ^15^N ratios in the hair segments. A total of 863 samples were analyzed using an Isoprime isotope ratio mass spectrometer (Isoprime Ltd, Cheadle Hulme, Stockport, UK) coupled to a CN elemental analyzer (Eurovector, Milan, Italy) with continuous flow. The natural abundance of ^15^N was expressed as δ^15^N (‰) = 1000 (*R*sample—*R*standard) / *R*standard where *R* = mass 29 / mass 28, and the standard had previously been calibrated against atmospheric N_2._ Atmospheric δ^15^N = 0‰, by definition. Samples were analyzed with reference gas calibrated against standards from International Atomic Energy Agency IAEA N1, N2 and US Geological Survey USGS 25, 26, and drift correlated using peach leaves from US National Institute of Standards and Technology (NIST) as internal standard, as in Kristensen et al. [26].

To test the potential impact of using agarose gel to fixate the guard hairs on their stable isotope ratios, we tested 5 replicates of guard hairs from the same individual with and without encapsulation in agarose gel. There was no significant effect of the agarose gel (t = 1.72, p-value = 0.16).

To aid the interpretation of the stable isotope ratios over time as indicator of muskox diets, we used earlier collected data on stable isotope ratios in muskox feces at Zackenberg from Kristensen et al. [26] and fecal samples from various seasons. Fecal samples were collected randomly within the Zackenberg valley with a minimum distance of 100m between each sampling. Feces from were collected in April (late winter), May (early spring), June (late spring), August (summer), and October (early winter). At least 30 separate fecal samples were collected from each season.

### Alignment of time series and data analysis

Many grass-fed animals exhibit a similar pattern of a seasonal variability in hair δ^15^N [7, 43], but there are usually large differences in amplitudes, which can be explained by animals utilizing different areas, where the variability in plant δ^15^N ratios depend on many different factors [43–45]. Yet, previous studies has shown that even socially unrelated individuals exhibit the same seasonal patterns [7]. Hence, in order to get a population level dietary signal focusing on seasonal patterns, the stable isotope sequences from the 10 individuals were first standardized within each individual (mean = 0, standard deviation = 1). We then used the mean of the 10 individuals as an expression of the population level stable isotope signature over time. We thereby assumed the variation in the hair growth rates to be low both within and between individuals [4, 7, 46].

To determine the temporal resolution of our 2 mm guard hair segments, we first used an autocorrelation function to detect periodicity in the dietary chronology, and hence to detect seasonal fluctuations in the diet. Assuming that summer diets are positively correlated in time, and summer diet and winter diet are negatively correlated, we then converted the periodicity to growth rates for one year (i.e. from summer diet (peak δ^15^N) to summer diet (peak δ^15^N)), and thus converted the 2 mm guard hair segments to an estimated time interval. To visually verify this, we aligned the stable isotope time series with local time series of air temperature, snow depth and meadow productivity (Normalized Vegetation Difference Index (NDVI)) with known time stamp. In doing so, we implicitly disregarded any temporal mismatch brought about by time needed for elemental incorporation into the hairs [4, 7], as well as hair remains not included due to shaving instead of hair pulling [4]. The alignment of time series, hence, only serves as a temporal yard stick [7].

As a general descriptor of the intra- and inter-annual variation in environmental conditions, we used the mean air temperature (°C) and mean snow depth (m), recorded hourly from an automatic weather station located centrally in the Zackenberg valley [36]. The Normalized Vegetation Difference Index (NDVI) was measured using a handheld CropCircle handheld system on a weekly basis during the snow free periods inside permanently monitored meadow plots located in the valley lowland at Zackenberg [47].

The effects of air temperature and snow depth and their interactions on the δ^15^N dietary signal over the time series was analyzed in a mixed first-order autoregressive model with individual as random factor (Proc Mixed, SAS 9.4). Model reduction was conducted by successively removing the non-significant (*P* > 0.05) model variables. NDVI was not included in the model, as the measurements are not continuous throughout the time series.

### Ethics statement

Capture and handling of muskoxen in this study followed the guidelines of the American Society of Mammalogists [48], and was approved the Government of Greenland (Permit number; j.no. G13-029).

10 adult muskox cows were sedated by approaching on foot and darted from a distance of approximately 30–50 meters using a CO2 driven dart gun (JM Special, DanInject, Børkop). Each cow was immobilized using of 2.0 mg Etorfin (Captivon 9,8 mg/ml Wildlife Pharmaceuticals South Africa), 30 mg Xylazine (Rompun dry substance, Bayer Healthcare), 0.3 mg Medetomidine (Zalopine 30 mg/ml, Orion Pharma) and 40 mg Ketamine (Ketaminol 100 mg/ml, MDS Animal Health). After immobilization, the animals were placed in sternal recumbancy and provided pure oxygen (Air Liquide 2 L/min), via a thin tube through the nostrils to the pharynges. Hair samples were cut from the rump region using an electric hair clipper. At all times, at least one person was monitoring breathing, anesthesia level, and belching. After samples were taken, the animal was given an antidote intramuscularly consisting of 50 mg Naltrexon (Trexonil 50 mg/ml Wildlife Pharmaceuticals) and 5 mg Atipamezole (Antisedan 5 mg/ml Orion Pharma). Within a few minutes after the antidote was given, the animal stood up and began moving towards its group. All handled animals experienced no trauma, and all of the animals have since moved around the area.

## Results

**Fig 1:**
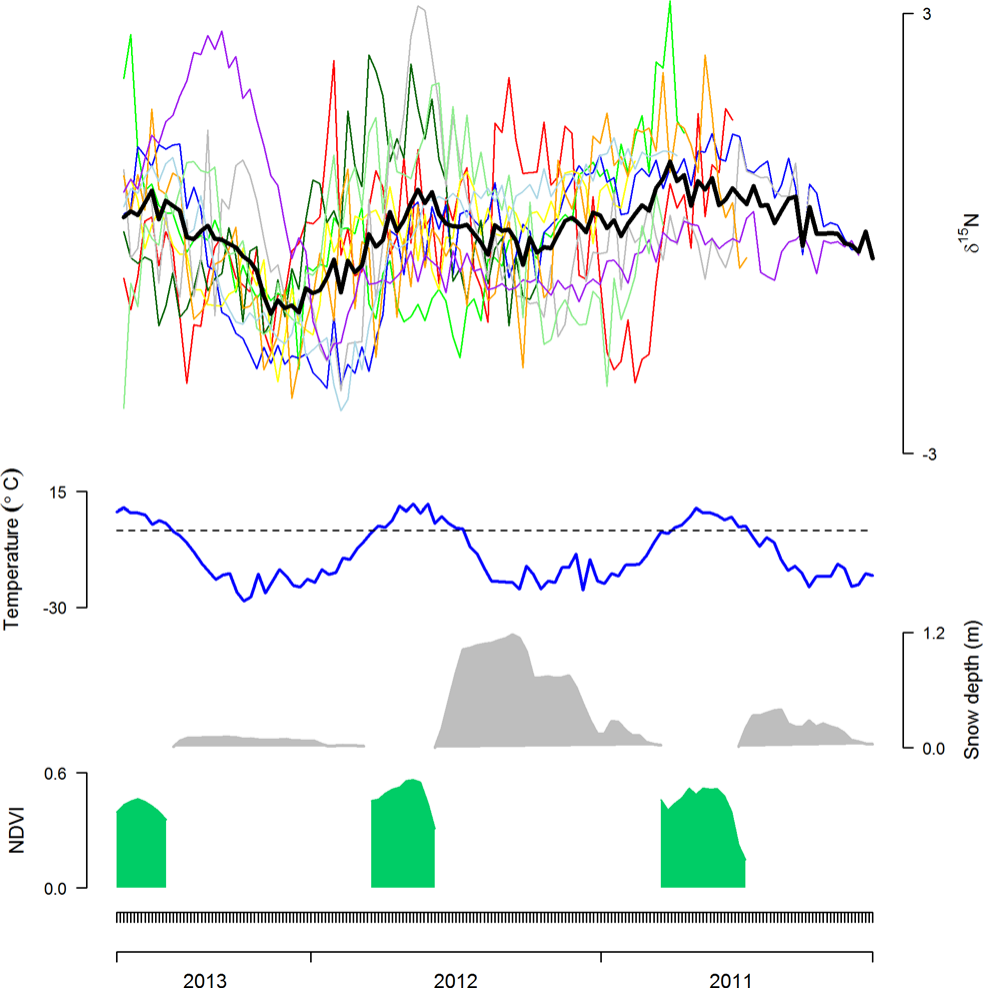
Muskox dietary history and ambient environment. Shown in the top is the muskox (*Ovibos moschatus*) dietary history inferred from the standardized nitrogen isotope ratios (δ^15^N) in guard hairs from 10 muskox cows and their mean (black line), covering approximately 2.5 years with a temporal resolution of app. 9 days. Below the stable isotope ratios are shown the ambient environmental conditions: Mean air temperature (°C), mean snow depth (m) and meadow productivity (NDVI) from the study area in the 9-day intervals. The guard hair dietary chronology matched the local environmental fluctuations, and included almost three full summer (high δ^15^N ratios) and winter periods (low δ^15^N ratios). Compared to summer diets, winter diets exhibit more pronounced inter-annual variation.

**Fig 2:**
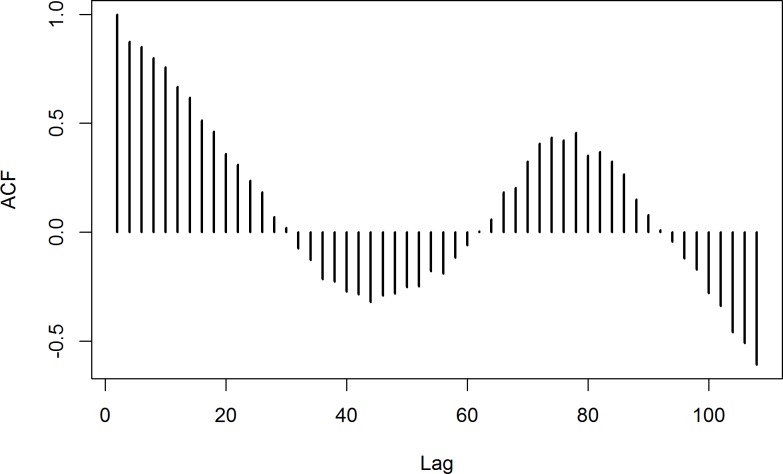
Autocorrelation function of the standardized δ^15^N ratios exhibiting clear periodicity. We assumed that summer diets were positively correlated in time, whereas summer diet and winter diets were negatively correlated, resulting in one year corresponding to 78mm of growth in the guard hairs.

**Fig 3:**
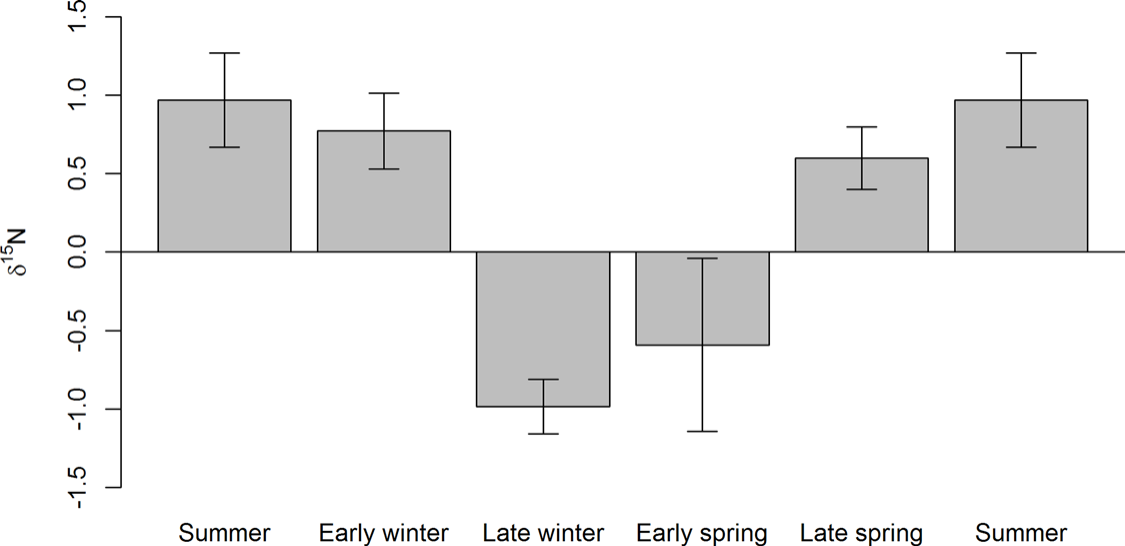
The stable isotope δ^15^N ratios in muskox feces. Feces were collected at Zackenberg throughout the year over multiple years, and used to support the interpretation of the stable isotope δ^15^N ratios in muskox guard hairs. The data are obtained from previous studies and over multiple years (2010–2013) [26].

The length of the guard hairs used in this study ranged from 12–22 cm. There was a large difference in the standardized δ^15^N ratios between the 10 individuals (Fig 1). Nonetheless, the time series of the *mean* standardized δ^15^N ratios varied from 0.98‰ to -1.10‰, and exhibited a clear cyclic pattern with varying amplitude (Figs 1 and 2). The autocorrelation function detected a clear periodicity, indicating app. 78mm of guard hair growth between each summer diet signal (Fig 2). When temporally aligned with the time series of environmental data from Zackenberg, the cyclic pattern matched the patterns in the temperature curve in particular, but also those of snow depth and plant productivity (Fig 1). The δ^15^N dietary time series was significantly positively correlated with the air temperature, negatively with the snow depth, and negatively with their interaction (Table 1). The autoregressive component in the model was significant (G_1_ = 754.0, P < 0.001), meaning that δ^15^N in the hair segment was affected by the former time step. Also, the effect of the random component (individual) was significant (G_1_ = 5.4, P = 0.020). Hence, it suggests that the seasonal pattern in the δ^15^N ratios indeed reflect dietary seasonality. This was further corroborated by the δ^15^N ratios in muskox feces, exhibiting a similar seasonal pattern of high δ^15^N ratios in summer and low δ^15^N ratios in winter (Fig 3).

**Table 1:** Summary statistics.

Variable	Coefficient	± S.E	DF	F value	P value
Air temperature (°C)	0.02479	0.003121	842	63.11	< .0001
Snow depth (m)	-0.2945	0.1251	821	5.55	0.0187
Temperature*Snow depth	-0.01788	0.008057	667	4.93	0.0268

Summary statistics for the mixed first-order model autoregressive model with individual as random factor. Both the autoregressive (G_1_ = 754.0, P < 0.001), and random component (G_1_ = 5.4, P = 0.020) were significant for the model.

The guard hairs provided a chronology of approximately 2.5 years with a temporal resolution of 9 days, and included almost three full summer- and three winter periods (Fig 1), spanning approximately January 2011 –September 2013. While the δ^15^N ratios in the summer periods (positive temperatures) were rather similar (Fig 1), the δ^15^N ratios in winter differed between the three winters (very low temperatures), and in particular between the winters of 2011/2012 and 2012/2013. Temperature-wise the two winters were similar, but differed markedly with respect to the amount of snow: The winter 2011/12 had large amounts of snow, whereas the winter of 2011/2012 almost had no snow on the ground (Fig 1).

## Discussion

Identifying the links between environmental conditions and their likely consequences for animal populations dynamics are of great interest in animal ecology [29]. These links are often mediated through dietary changes, both intra- and inter-annually. Stable isotopes offers a promising tool for tracking dietary changes, and hence, could be a valuable tool for species monitoring [10–12]. Stable isotope analyses have previously been used to trace the dietary history of animals, for instance those living in environments dominated by tropical grasses (C_4_ plants) [5, 6, 9, 49], or in Arctic mammals with terrestrial and marine food sources [50]. Recently, Cerling et al. [5] linked the seasonal changes in elephant diet to changes in the ambient environmental conditions through sequential analyses of stable isotopes in tail hairs. Here, we successfully used sequential stable isotope analyses of δ^15^N ratios in muskox guard hairs to recreate an approximately 2.5 year’s dietary chronology with a high temporal resolution of 9 days. Specifically, we reconstructed the dietary signal of almost three full summer- and three winter seasons in high arctic Greenland, spanning from approximately January 2011 to September 2013 (Fig 1).

Our results indicated a considerable variation in the δ^15^N ratios at the individual level (G_1_ = 5.4, P = 0.020). The high individual variation was not surprising, as even mammalian herbivores eating identical diets can have hair δ^15^N ratios that differ up to 3.6‰ [51]. Plant δ^15^N ratios can vary greatly due to a number of physiological and abiotic factors [52], and even within a small area, there can be a variation of over 10‰ in plant δ^15^N [34, 52, 53]. Plant δ^15^N ratios could also be regulated by nitrogen availability, which may be influenced by the defecation and urination of these large animals [54]. However, muskoxen generally feed less in areas where most of the defecation takes place, and vice versa [54, 55]. Further, in wild roaming herbivores, animals in a population often utilize different areas, where the variability in plant δ^15^N ratios depend on different factors such as soil conditions, nitrogen availability, dominant forage type, nitrogen recycling within a plant, climate, altitude, and distance from sea [43–45], thus creating individual variation within a population. Yet even socially unrelated individuals at the population level exhibit the same seasonal patterns in their diet [7, 43]. Indeed, despite our large individual variation, there was a clear seasonal dietary pattern at the population level (Fig 1). Muskoxen exhibit large seasonal patterns in body condition [21, 23], which is linked to the quality and quantity of the forage in the strongly seasonal, high latitude environments where they roam [19, 20]. In addition, muskox forage is mainly dominated by graminoids in summer, and willow twigs in winter [19, 26, 27, 38]. As these plant groups have different δ^15^N ratios in the Zackenberg valley, with higher δ^15^N in graminoids (average δ^15^N 1.5‰) than in willows (average δ^15^N -4.7‰) [26, 34], we expected to find a strong seasonal pattern in the δ^15^N hair chronology, as well as in the collected feces, reflecting the seasonal changes in diet. Indeed, both the feces and the reconstructed muskox diet from guard hairs exhibited a strong seasonality in the δ^15^N ratios, with summer periods (i.e. the warm periods; Fig 1) values generally having higher values than the winter periods (i.e. cold and snow-covered periods) (Figs 1–3); a pattern that was verified in the statistical analyses (Table 1). This has several implications. First, our temporal alignment was indeed adequate, and each guard hair segment thus corresponds to a nine day period (growth rate of 0.2 mm/day, growing app. 78 mm/year). Second, as the δ^15^N ratio and the temperature curves did not slide apart (Fig 1), the growth rate of muskox guard hair from the rump region indeed seems to be constant across the year [42]. Finally, it suggests that the seasonal pattern in the δ^15^N ratios indeed reflect dietary seasonality, reflecting a higher consumption of graminoids in summer. The latter was supported by the seasonal pattern in the δ^15^N ratios in fecal samples, collected throughout the year at Zackenberg, exhibiting the same pattern as the guard hairs of high δ^15^N ratios in summer and low δ^15^N ratios in winter (Fig 3). A similar, tight linkage between seasonal changes in climate and biomass to dietary signals in hair chronologies was reported by Cerling et al. [5] for African elephants, and Zazzo et al. [43] for sheep. Hence, our results strongly support that sequential stable isotopes in hair constitutes a strong tool to link environmental conditions to the dietary record of animals. The question is then whether this tool can be used to unravel ecological information, and ultimately the causes of muskoxen population level changes?

The muskox population dynamics at Zackenberg is largely driven by changes in calf recruitment, which have experienced large variations throughout the years [35]. Changes in calf recruitment may be linked to the nutritional status of the population during the rutting season [22, 25]. As graminoids have high crude protein contents and a high digestibility [20], access to graminoids during summer and into the autumn is likely to play a major role in replenishing the body stores that are crucial for winter survival and calf production [29]. In our study period, the inter-annual variation in muskox summer diet was small compared to winter, and the δ^15^N ratios were always high in summer, suggesting a relatively uniform summer diet dominated by graminoids, irrespective the inter-annual variation in environmental conditions and plant productivity. This may reflect that availability of summer forage in general, and graminoids in particular, does not seem to be limited at Zackenberg [54]. Small inter-annual variation in the summer forage availability, as indicated by the low inter-annual variation in NDVI (Fig 1), are thus unlikely to induce large variations in the summer dietary signal in the guard hairs. Instead this is more likely to be affected by the length of seasons and winter forage availability and quality [56, 57]. The onset of snow significantly starts the decline of the δ^15^N to a lower winter isotope ratio in the guard hairs, effectively determining the length of the summer season (Table 1, Fig 1).

In contrast to the summer diet, the winter diet of muskoxen differed markedly between the winter seasons included in our dietary chronology (Fig 1). The inter-annual variation in winter diet appeared to be linked to differences in snow depth rather than temperature, and compared to the snow-rich year, the snow-poor year resulted in the largest seasonal differences in δ^15^N (Fig 1). In winter, most forage is unavailable for muskoxen [58], and muskoxen rely heavily on their body storages [21, 23], while supplementing with intake of plants like willows, which can be accessed under the snow or on barren and windswept areas uphill [19, 20]. However, muskoxen have a fairly low snow depth threshold of about 23 cm, and snow depth exceeding the threshold makes it almost impossible for the muskoxen to access the forage below [59]. Therefore, we expected severe (i.e. snow-rich) winters to result in starvation periods for the muskoxen, forcing the animals to rely more on the body reserves, built up on graminoids in summer and autumn, resulting in increased δ^15^N ratios (i.e. graminoid-dominated δ^15^N signal), even in winter, compared to snow-poor winters. In addition, catabolism of body stores may in itself increase δ^15^N ratios [11, 28, 60, 61], as catabolism results in the recycling of body protein, producing a trophic level effect within the animal itself. To date, an increase in δ^15^N ratios during starvation periods has been observed in reindeers, muskoxen, humans, arthropods, fish, and birds [28, 61–66]. Indeed, our results showed that the δ^15^N ratios in hair from the snow-rich winter of 2011/12 had markedly higher δ^15^N ratios than the snow-poorer winters of 2012/13 and 2010/11 (Fig 1). This indicates that the muskoxen were experiencing prolonged periods of starvation. Additionally, the strong isotopic signal from willows in the snow-poor winter of 2012/13, suggests that the plant isotopic signal indeed is evident and stronger than that of the body stores when winters were favorable, despite that muskoxen in such winters still may undergo periods of starvation while relying on their body stores [21, 23].Therefore, the increased winter δ^15^N ratios in snow-rich winters strongly suggests that muskoxen have very limited access to willow forage, resulting in muskoxen relying even more on their body reserves, resulting in increased starvation periods and weight loss [61]. Thus, it would also reflect that body proteins are increasingly being used for maintaining body condition of the cow instead of being used for calf growth [28].

The link between high winter δ^15^N ratios and catabolism of body stores, in particular maternal body proteins, suggests that high δ^15^N ratios in winter may be a suitable indicator of poor calf production. Such a relationship has previously been established for both reindeer and muskoxen using stable isotopes from excreta [12, 28, 29, 60, 62]. The results of the current study highlight the potential of using stable isotopes in hair to monitor muskox populations, if one can establish the link between the dietary signal in muskox guard hairs, and vital population demographic parameters such as calf recruitment. At Zackenberg, calf recruitment has varied a lot throughout the years, which is mainly attributable to variation in snow conditions, but likely also pathogens [35]. Noteworthy, is however that the calf recruitment at Zackenberg following the three winters included in our dietary chronology and the δ^15^N exhibited a consistent pattern of high calf recruitment in summer 2013, low in summer 2012, and intermediate in summer 2011, with 68%, 8%, and 26% of the cows having calves in the summer of 2013, 2011, and 2010, respectively (compare to Fig 1) [35]. Nonetheless, any pattern over shorter time should warrant caution, and longer time series is needed to confirm such links.

We have successfully reconstructed, for the first time, the dietary record of muskoxen with a high temporal resolution. The muskox diet exhibited a strong seasonality, and could be linked to changing intra- and inter-annual environmental conditions. Being able to understand the link between environmental conditions and animal diets, and thus ultimately to population dynamics, would greatly increase our ability to inform proper conservation and management initiatives. Although only three winter periods are included in our dietary chronology and any patterns warrant caution, our study indicates a link between δ^15^N ratios in winter hair and calf recruitment in muskoxen the following summer. Our study opens the field for further studies and longer chronologies to test such links. Our results have shown that muskox guard hairs can be used as a dietary archive, and that stable isotope ratios in the hair may be linked to vital population demographic parameters. The method of sequential stable isotope analysis of guard hairs thus constitutes a promising candidate for population-level monitoring of animals in remote regions, such as the Arctic.

## Supporting Information

S1 File Stable isotope dataset. (XLSX)
